# Feasibility of Continual Deep Learning-Based Segmentation for Personalized Adaptive Radiation Therapy in Head and Neck Area

**DOI:** 10.3390/cancers13040702

**Published:** 2021-02-09

**Authors:** Nalee Kim, Jaehee Chun, Jee Suk Chang, Chang Geol Lee, Ki Chang Keum, Jin Sung Kim

**Affiliations:** Department of Radiation Oncology, Yonsei Cancer Center, Yonsei University College of Medicine, Seoul 03722, Korea; nalkim@yuhs.ac (N.K.); cjhsmile@yonsei.ac.kr (J.C.); changjeesuk@yuhs.ac (J.S.C.); cglee1023@yuhs.ac (C.G.L.); kckeum@yuhs.ac (K.C.K.)

**Keywords:** head and neck cancer, deep learning, auto segmentation, artificial intelligence, adaptive radiation therapy

## Abstract

**Simple Summary:**

We analyzed the contouring data of 23 organs-at-risk from 100 patients with head and neck cancer who underwent definitive radiation therapy (RT). Deep learning-based segmentation (DLS) with continual training was compared to DLS with conventional training and deformable image registration (DIR) in both quantitative and qualitative (Turing’s test) methods. Results indicate the effectiveness of DLS over DIR and that of DLS with continual training over DLS with conventional training in contouring for head and neck region, especially for glandular structures. DLS with continual training might be beneficial for optimizing personalized adaptive RT in head and neck region.

**Abstract:**

This study investigated the feasibility of deep learning-based segmentation (DLS) and continual training for adaptive radiotherapy (RT) of head and neck (H&N) cancer. One-hundred patients treated with definitive RT were included. Based on 23 organs-at-risk (OARs) manually segmented in initial planning computed tomography (CT), modified FC-DenseNet was trained for DLS: (i) using data obtained from 60 patients, with 20 matched patients in the test set (DLSm); (ii) using data obtained from 60 identical patients with 20 unmatched patients in the test set (DLSu). Manually contoured OARs in adaptive planning CT for independent 20 patients were provided as test sets. Deformable image registration (DIR) was also performed. All 23 OARs were compared using quantitative measurements, and nine OARs were also evaluated via subjective assessment from 26 observers using the Turing test. DLSm achieved better performance than both DLSu and DIR (mean Dice similarity coefficient; 0.83 vs. 0.80 vs. 0.70), mainly for glandular structures, whose volume significantly reduced during RT. Based on subjective measurements, DLS is often perceived as a human (49.2%). Furthermore, DLSm is preferred over DLSu (67.2%) and DIR (96.7%), with a similar rate of required revision to that of manual segmentation (28.0% vs. 29.7%). In conclusion, DLS was effective and preferred over DIR. Additionally, continual DLS training is required for an effective optimization and robustness in personalized adaptive RT.

## 1. Introduction

The standard treatment for head and neck (H&N) cancer entails tri-modality therapy, including surgery, chemotherapy, and radiotherapy (RT). Particularly, intensity-modulated radiation therapy (IMRT) could achieve a homogeneous dose distribution in the target area, minimizing the radiation dose to normal organs. Moreover, the evolution of image-guided RT has led to adaptive RT (ART), which accounts for anatomical changes arising from weight loss or tumor regression during RT, aiming to provide accurate and precise dose delivery [1]. Interestingly, planning computed tomography (CT) data of the same patient with temporal changes during RT is used for each ART. Detecting changes between initial planning CT and adaptive CT and dynamic adaptation is required for the optimized adaptive RT. 

In accordance with technical developments in RT, the planning process becomes complicated and important. After planning a computed tomography (CT) acquisition, the segmentation of the target area (gross tumor volume, clinical target volume, and planning target volume) and organs-at-risk (OARs), called contouring, is needed to obtain 3-dimensional volumetric information for planning standardization and quality assessment. The planning quality as well as the time expended on the contouring process largely depend on the personal experience of physicians or technicians. Despite several consensus guidelines for contouring [2,3], both inter-observer and intra-observer variability remain an issue for standardization and qualified planning [4,5,6,7,8,9]. In addition, the H&N region includes more than 20 OARs, requiring more than 1–2 h for contouring per patient. Consequently, a survey discovered that contouring for the H&N region is more difficult for physicians than it is for other sites treated with RT [10,11]. Moreover, the burden of contouring hinders real-time or frequent ART in H&N cancer. Therefore, auto-segmentation in the H&N region is essential for standardization and efficiency in treatment planning. 

Various auto-segmentation tools have been developed: deformable image registration (DIR), atlas-based auto-segmentation, and recent deep learning-based segmentation (DLS) [12]. Both DIR and atlas-based auto-segmentation have been widely implemented, but they have several limitations in clinical utilization [13,14]. Based on substantial artificial intelligence research, several reports suggested DLS as a promising method for segmentation in the H&N region. Most previous reports evaluated 3–5 OARs with the Dice similarity coefficient (DSC) range of 0.37–0.99 [12]. However, for further clinical applications in the H&N region, an auto-segmentation of multiple OARs with up to 20–25 subsites is essential. 

Besides, the major hurdle for developing clinically feasible DLS model starts from the amount of training samples [15,16]. Although the issue of overfitting is considered as a challenge in investigations regarding deep learning algorithm, the intentional overfit using patient-specific prior information could be considered to improve the generalizability of DLS for clinical applications during ART. Patients candidates for ART have large amount of previous data for DLS including diagnostic CT, initial planning CT, and even kilo-voltage/mega-voltage cone-beam CT. In other words, the ART-optimized DLS needs to be optimized to produce the overfitted performance for the specific patient with prior information rather than generalized performance for future patients. In this context, a continual training with initial data for specific patient is considered for DLS in ART of H&N cancer. Yet, there is no report regarding continual training for DLS concerning ART for H&N cancer. That is, whether DLS for ART needs re-training based on individual initial planning data points is still unclear. To address the aforenoted limitations, in the current study, we evaluate the feasibility of DLS and the ideal training set for DLS in ART for the H&N region.

## 2. Materials and Methods

### 2.1. Patients

Patients with H&N cancer treated via RT were included in this study. The inclusion criteria for the entire cohort were as follows: (1) patients with pharyngeal (nasopharynx, oropharynx, and hypopharynx) cancer, (2) patients treated with definitive RT, (3) patients who underwent adaptive planning CT (aCT), and (4) patients with available contrast-enhanced planning CT for both primary CT (pCT) and aCT. We excluded patients who had a history of surgery in the H&N region and whose planning CT was performed with a more than 3 mm slice thickness. All planning CT scans (either based on Aquilion TSX-201A, Toshiba, Tokyo, Japan, or Somatom Sensation Open Syngo CT 2009E, Siemens, Munich, Germany) were performed using a thermoplastic immobilization system (Type-S; Medtec, Alton, IA, USA) with a slice thickness of 3 mm. We routinely performed pCT 2 weeks prior to RT and aCT 15 fractions after the initiation of RT. The median interval between aCT and pCT was 36 days (range: 29–43). This study was approved by the institutional review board of the Yonsei Cancer Center (No. 4-2020-0001), and the protocol conformed to the ethical guidelines of the 1975 Declaration of Helsinki. Owing to the retrospective nature of this study, the need for informed consent was waived. Because we only included patients who had already completed the scheduled treatment, the contours generated via DLS or DIR were never used for actual treatment planning.

### 2.2. Manual Segmentation

The OARs were manually contoured by a single radiation oncologist according to the consensus guidelines [3]. We included 23 OARs categorized into 4 groups as follows: (a) central organs, (b) bony structures, (c) glandular structures, and (d) optic apparatus (Table S1). All contours were generated using MIM Maestro 6.7 (MIM Software Inc., Cleveland, OH, USA).

### 2.3. Deep Learning-Based Segmentation: Training Set and Preprocessing

In total, 100 patients were randomly selected: pCT images of 100 patients were employed as the training set, and the aCT images of the 20 patients were selected as the test set. 

**Figure 1:**
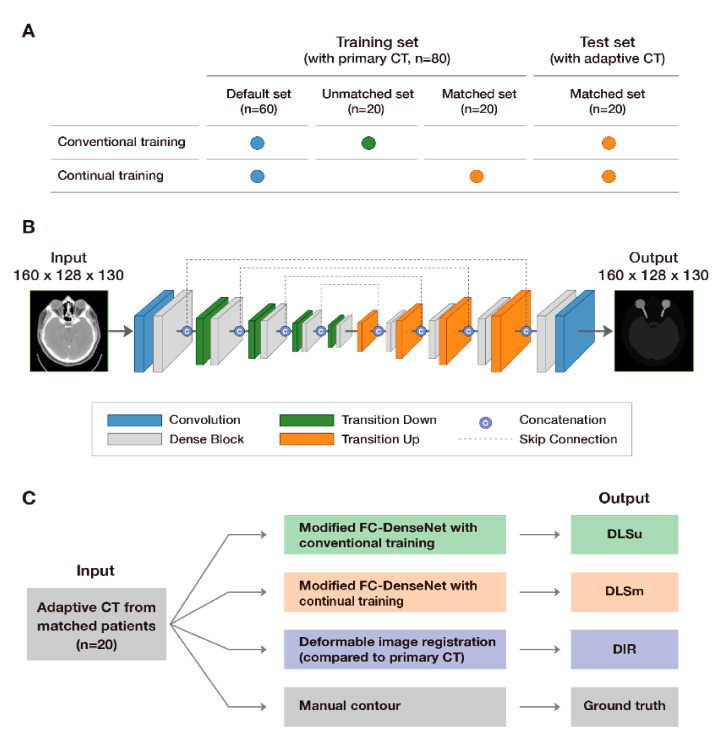
Study scheme: allocation of input data (**A**), architecture of modified FC-DenseNet (**B**), and model validation (**C**).

Two different training datasets including data regarding 80 patients were employed for DLS: a matched training set (continual training) which consists of pCT of 60 patients including pCT data from 20 patients in the test set and an unmatched training set (conventional training) which consists of pCT images corresponding to the same 60 patients with pCT data regarding 20 different patients from the test set. (Figure 1A). Table S2 presents the baseline characteristics of the training and test sets. 

Because each original planning CT image had a different resolution, we normalized the images to 1.0 × 1.0 × 3.0 mm^3^ for the robustness of DLS. Next, we cropped the planning CT images of 120 patients with a global field-of-view volume size of 320 × 256 × 130, which encompassed all regions-of-interest (ROIs) of OARs. Lastly, we adjusted the window level, based on the Hounsfield unit, from [–100, 300] to [–1.0, 1.0]. This was performed to improve relative contrast and normalize all input data to the same range.

### 2.4. Deep Learning-Based Segmentation: Two-Step Segmentation and Network Architecture

We performed DLS in two steps, as we previously reported (localization and ROI-specific segmentation), with a modified fully convolutional DenseNet [17] (Figure 1B). The DenseNet was implemented using TensorFlow in Python. In the localization process, down-sampling by half in both the x and y directions with the reduction of image resolution was performed: the final input images had a size of 160 × 128 × 130 with a resolution of 2.0 × 2.0 × 3.0 mm^3^. Subsequently, we separated each OAR simultaneously via multilabel segmentation concerning each ROI in the preprocessed images. In the second step, we carried out single-label segmentation for each OAR from the ROIs in the first step. Specifically, we calculated the middle point of each predicted volume in the first step. From the shared middle point, ROIs for each OAR that have minimal margin outside the volume were determined based on the pre-set size of ROIs in x-, y-, and z-axis (e.g., 144 × 176 × 48 for thyroid). Finally, single-label segmentation was performed in those ROIs during the second step.

The resolution of input data could be preserved without down-sampling (1.0 × 1.0 × 3.0 mm^3^) because we used a cropped ROI for each OAR. We modified a fully convolutional DenseNet in a three-dimensional manner. The architecture consisted of a dense block for preserving high-level features. Furthermore, the number of layers in each block was [3, 4, 4, 5, 7], and the growth rate and learning rate were 12 and 0.0005, respectively. The number of epochs was 250 and 200 for first and second step, respectively. We used the Adam as an optimizer and considered dual cross entropy as a loss function [18]. Moreover, there were four transition down and up blocks with skip connections from the down-sampling path to the up-sampling path as concatenations of the feature maps. The model was trained with a batch size of 1 owing to the memory usage entailed in three-dimensional segmentation.

### 2.5. Deformable Image Registration

We used a commercially available deformable registration software provided by MIM to perform intensity-based DIR using a free-form deformation [19,20]. The deformation was based on the demons optical flow algorithm. Regularization prevented tears and folds in the deformation field following the optimization performed via modified gradient descent. The process was performed by a blinded physician according to the standard process of ART. Contours were transferred from the pCT images of 20 patients to the corresponding aCT images of the 20 patients (test set).

### 2.6. Quantitative Evaluation

The computer-generated contours (*C*) obtained via conventional training of DLS on the unmatched training set (DLSu), continual training of DLS on the matched training set (DLSm), and deformable image registration from pCT (DIR) were compared with those obtained via manual segmentation (*M*, Figure 1C). The comparison was performed quantitatively based on similarity metrics, classic measurements, and distance measurements.

(1) Similarity metrics: The volumetric DSC calculates the spatial overlap between two binary images [21]:
  $$\mathrm{DSC}=\frac{2\left|C\cap M\right|}{\left|C\right|+\left|M\right|}.$$
(2) Classic measurement: False-positive DSC (FPD) and false-negative DSC (FND) calculate the falsely segmented and detected pixels, respectively [22]:
  $$\mathrm{FPD}\;=\frac{2\left|C\cap M^{-}\right|}{\left|C\right|+\left|M\right|}$$
  $$\mathrm{FND}\;=\frac{2\left|C^{-}\cap M\right|}{\left|C\right|+\left|M\right|}.$$
(3) Distance measurements: In both 95th percentile Hausdorff distance (HD) [23] and mean surface distance (MSD) calculation, the value of each voxel is the Euclidean distance in millimeters from each surface voxel of volume *C* to the nearest surface voxel of volume *M*. HD and MSD measure the distance and the mean of the absolute values of the surface distance between *C* and *M*, respectively:
  $$\mathrm{HD}\;=\;\mathrm{percentile}\;\left(Vector_{C,M}\cup Vector_{M,C},\;95^{th}\right)$$
  $$\mathrm{MSD}\;=\frac{1}{2}\;\left(Vector_{C,M}+Vector_{M,C}\right).$$

### 2.7. Subjective Evaluation

A Turing test that evaluates clinical usability was performed for a subjective evaluation of three contouring results [24]. All 26 observers from 3 different institutions (including 8 certified radiation oncologists, 5 medical physicists, 5 certified radiologists, 4 dosimetrists, and 4 residents) were blindly presented with random three-dimensional images for 9 OARs from the test sets (spinal cord, esophagus, oral cavity, pharynx, larynx, mandible, left parotid gland, right submandibular gland, and thyroid); such images are generally employed in routine RT planning for the H&N region. An example of the Turing test is available at https://forms.gle/uf7sXvKu5h51eCmd7 (accessed on: 12 December 2020). The following questions were provided to each observer in 198 scenarios, and the details regarding each question were adopted from a previous report [13]:

(1) Discrimination of a single contour from M and C (DLSu, DLSm, and DIR) concerning whether the contouring was performed by a human or a computer.
(2) Comparison between M vs. DLSm, DLSm vs. DLSu, and DLSu vs. DIR, respectively.
(3) Quality assurance, for review purposes, of a single contour from *M* and *C* (DLSu, DLSm, and DIR). Major error was defined as subjective assessment for difference more than 10% of single contour.

This study did not analyze the consistency of the assessment by observers (either intra- or interobserver) because this study mainly aimed to identify the optimal training set for ART preliminarily. A further investigation can be conducted with a multi-institutional dataset.

### 2.8. Contouring Time

For assessing the efficacy of DLS, we recorded the time to produce *M*, DLSu, and DLSm for the entire 23 OARs. Only time for running each built network in 20 patients of test set was recorded for DLS and time spent for data reading, writing, and preprocessing was not considered.

### 2.9. Statistical Analysis

After evaluating a normalized distribution via the Shapiro–Wilk test, we performed pairwise t-tests to compare DSC, FPD, FND, HD, and MSD. Because there are three segmentation methods (i.e., DLSm, DLSu, and DIR), a Bonferroni correction was adopted with an alpha value of 0.05/3 (0.017): the null hypothesis was rejected if *p* < 0.017, and the results were considered statistically significant. All statistical analyses were performed using R (version 3.6.3; R Foundation for Statistical Computing, Vienna, Austria).

## 3. Results

### 3.1. Baseline Information

No significant differences with regard to sex, primary tumor site, or T and N categories were observed between the matched and unmatched training sets and between the training and testing cohorts (Table S2). Furthermore, the volumes of most OARs remained constant between pCT and aCT, except for glandular structures (Table S3): there was a 10% volume reduction in glandular structures, especially the parotid and submandibular glands.

### 3.2. Quantitative Evaluation

#### 3.2.1. Overall Performance

**Figure 2:**
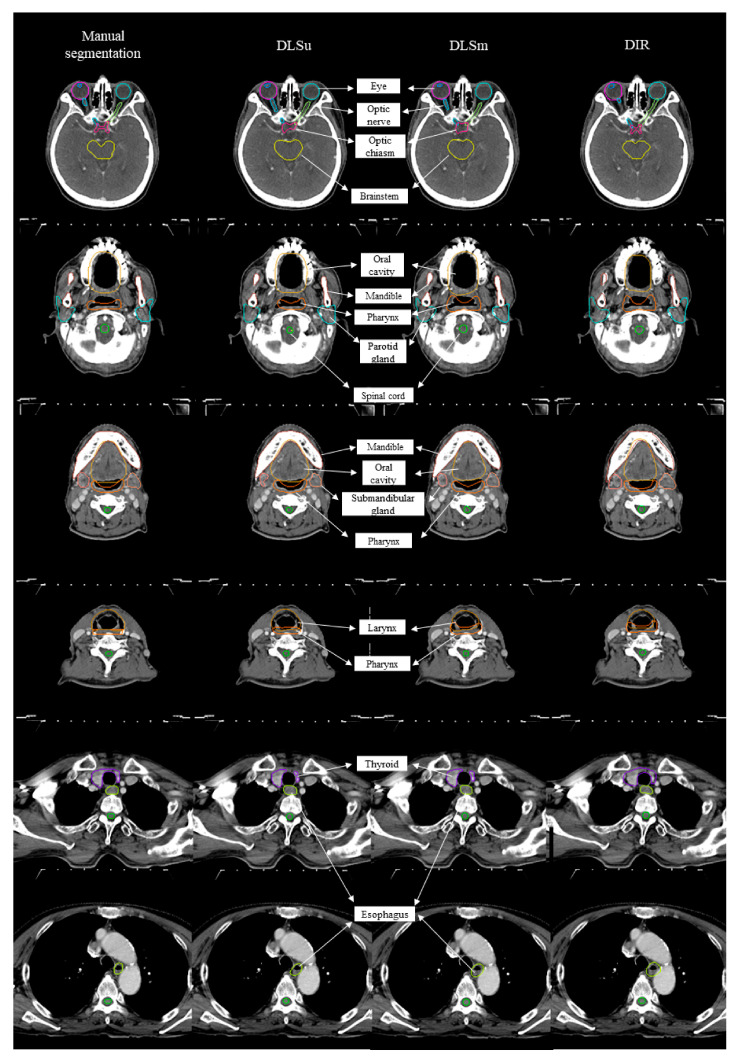
Examples of manual contour, deep learning-based segmentation based on the unmatched training set (DLSu) and matched training set (DLSm) and deformable image registration (DIR).

**Figure 3:**
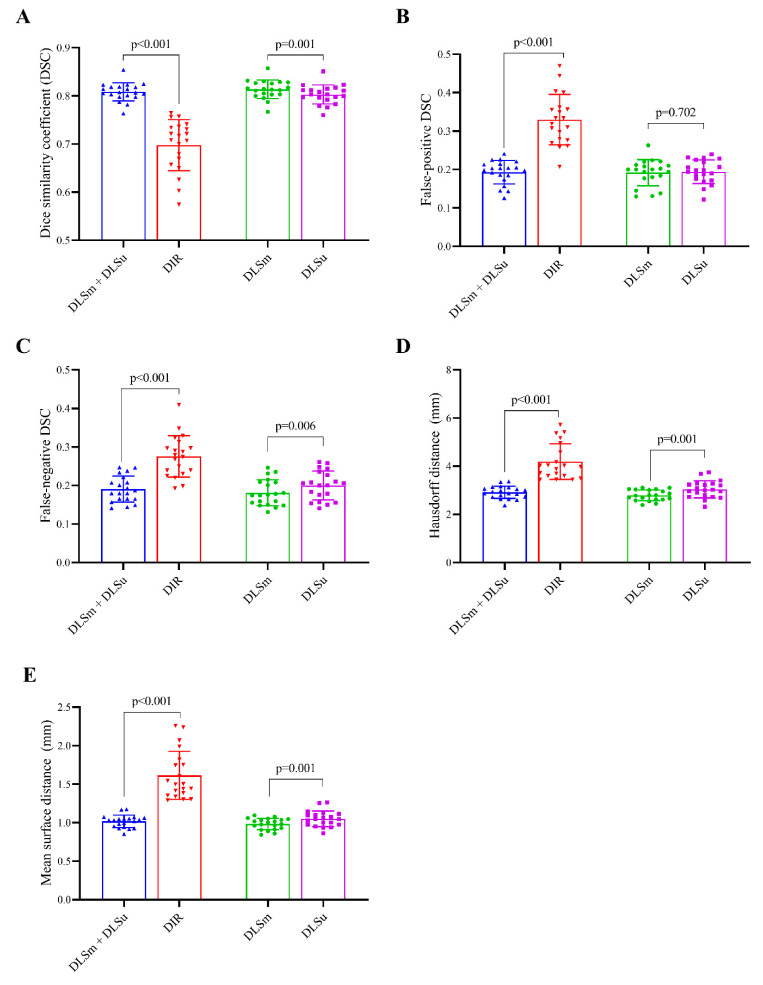
Median and interquartile range of average (**A**) Dice coefficient (DSC), (**B**) false positive Dice coefficient (FPD), (**C**) false negative Dice coefficient (FND), (**D**) Hausdorff distance (HD), and (**E**) Mean surface distance. Footnotes: *DLSm + DLSu is defined as the average value of DLSm and DLSu*, and mean surface distance (MSD). Abbreviations: DLSu, deep learning-based segmentation using the unmatched training set; DLSm, deep learning-based segmentation using the matched training set; DIR, segmentation from deformable image registration.

Figure 2 displays an example of DLSm, DLSu, and DIR. The averages for all tested values are summarized in Figure 3 and Table 1 and Table 2. The proposed DLS, irrespective of the training cohort, exhibited a better overall agreement with M than that shown by DIR, as evidenced by an increased mean DSC value (0.81 ± 0.02 vs. 0.70 ± 0.05, Figure 3A) with a reduced mean FPD (0.19 ± 0.03 vs. 0.33 ± 0.07, Figure 3B) and mean FND (0.19 ± 0.04 vs. 0.28 ± 0.05, Figure 3C). The HD and MSD values were also lower in the case of DLS compared with those exhibited by DIR (all *p* < 0.017, Figure 3D,E). Regarding the training set, DLSm exhibited minimally improved performance over that of DLSu, but the improvement was statistically significant (*p* < 0.017); here, the mean DSC increased from 0.80 ± 0.02 (DLSu) to 0.83 ± 0.02 (DLSm) with a significant reduction in FND (0.18 ± 0.03 vs. 0.20 ± 0.04, Figure 3C) rather than FPD (0.19 ± 0.03 vs. 0.19 ± 0.03, Figure 3B). In addition, DLSm minimally but statistically significantly reduced the distance between the automated and manual segmentation compared with that exhibited by DLSu (HD, 2.79 ± 0.22 vs. 3.04 ± 0.3; MSD, 0.98 ± 0.07 vs. 1.05 ± 0.10, Figure 3D,E).

#### 3.2.2. Central Organs

DLS resulted in better segmentation than DIR, especially for the spinal cord, pharynx, and larynx (*p* < 0.017); the largest difference in DSC was observed in the spinal cord (0.82 ± 0.04 vs. 0.67 ± 0.16), followed by the pharynx and larynx. In addition, DLS exhibited a significantly lower FPD compared with that exhibited by DIR with regard to the spinal cord (0.16 ± 0.10 vs. 0.33 ± 0.21), and HD and MSD exhibited by DLS were statistically different from those exhibited by DIR with regard to the spinal cord, pharynx, and larynx (*p* < 0.017). We did not observe a difference between DLSm and DLSu regarding DSC, FPD, FND, HD, and MSD. The detailed metrics for central organs are presented in Table 1 and Table 2 and Figure S1.

#### 3.2.3. Bony Structures

The quality of DLS and DIR in bony structures (i.e., the cochlea and the temporomandibular joint), except for the mandible, was barely satisfactory, with a mean DSC of 0.74 and 0.69, respectively. In the subgroup analysis of the mandible, the DSC of DLS was significantly higher than that of DIR (0.95 ± 0.01 vs. 0.85 ± 0.09), with a significant reduction in FPD (0.04 ± 0.02 vs. 0.15 ± 0.10); furthermore, HD also decreased from 3.55 ± 2.64 (DIR) to 1.28 ± 0.32 (DLS). There was no significant difference in the accuracy of bony structures between DLSm and DLSu. The detailed metrics for bony structures are presented in Table 1 and Table 2 and Figure S2.

#### 3.2.4. Glandular Structures

For all glandular structures, DSC, FND, and MSD exhibited by the DLS showed a salutary improvement over those exhibited by DIR (*p* < 0.017), with the largest difference being for the right submandibular gland, for which DSC increased from 0.71 ± 0.09 (DIR) to 0.88 ± 0.04 (DLSm). In addition, DLSm achieved a DSC superior to that obtained by DLSu in the parotid gland (mean DSC: 0.87 ± 0.03 vs. 0.85 ± 0.04, respectively) and the submandibular gland (mean DSC: 0.87 ± 0.04 vs. 0.82 ± 0.08) with significant improvement in FND, HD, and MSD (*p* < 0.017). Moreover, DLSm for the submandibular gland also exhibited a lower FPD compared with that exhibited by DLSu. The detailed metrics for glandular structures are presented in Table 1 and Table 2 and Figure S3. 

#### 3.2.5. Optic Apparatus

Although there was a significant improvement in performance regarding DLS compared to DIR concerning all substructures of the optic apparatus, DLS exhibited a low DSC of 0.52 ± 0.17 for the optic chiasm. Both DLSu and DLSm exhibited similar accuracies regarding DSC, FND, FPD, HD, and MSD for all structures of the optic apparatus. The detailed metrics for optic apparatus are presented in Table 1 and Table 2 and Figure S4.

### 3.3. Time

**Figure 4:**
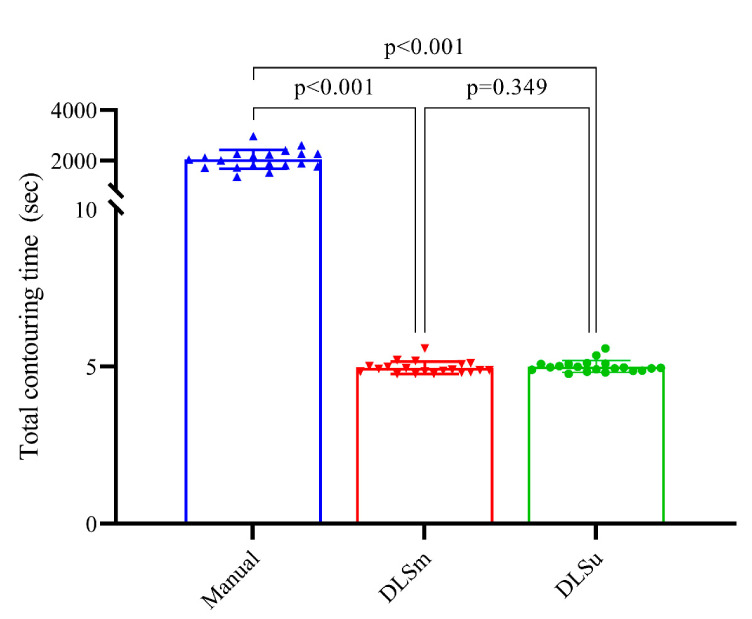
Mean and standard deviation of contouring time for 23 structures. Abbreviations: DLSu, deep learning-based segmentation using the unmatched training set; DLSm, deep learning-based segmentation using the matched training set.

There was a significant time reduction regarding DLS compared to M for contouring 23 OARs (*p* < 0.001). The mean time values spent for M, DLSu, DLSm were 2051.20 ± 374.51, 5.01 ± 0.19, and 4.96 ± 0.29, respectively (Figure 4). In addition, the processing time for DLSu and DLSm was comparable (*p* = 0.349). 

### 3.4. Subjective Evaluation

**Figure 5:**
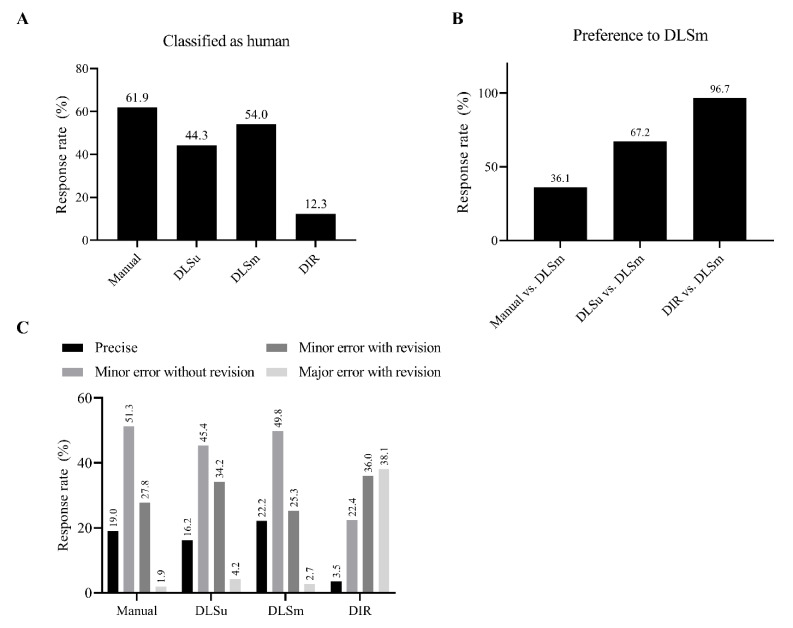
Subjective evaluation using the Turing test. The rate of discrimination of a single contour as having been generated by a human or a computer (**A**); comparison between two contours (**B**); quality assurance for review purposes of a single contour (**C**). Abbreviations: DLSu, deep learning-based segmentation using the unmatched training set; DLSm, deep learning-based segmentation using the matched training set; DIR, segmentation from deformable image registration.

Overall, 38.1% of M was misclassified as C; results of DLSm were more frequently considered human-generated contours than those of DLSu (54.0% vs. 44.3%, Figure 5A). For individual OARs, the rate of classification was similar between DLS and M for the right submandibular gland (DLS vs. M; 62.0% vs. 58.7%, Figure S5) and the oral cavity (63.3% vs. 64.2%). More than 50% of participants discriminated DLS as M for the submandibular gland, thyroid, mandible, oral cavity, pharynx, esophagus, and spinal cord in the case of DLSm and for the submandibular gland and oral cavity in the case of DLSu. A significant difference of more than 10% between DLSu and DLSm was observed for the larynx, pharynx, esophagus, and spinal cord. 

Although M was more frequently preferred over DLSm (63.9% vs. 36.1%, Figure 5B), DLSm was significantly preferred over DLSu (67.2% vs. 32.8%) and DIR (96.7% vs. 3.3%). DLSm showed a similar rate of preference for the spinal cord, mandible, thyroid, and submandibular gland when compared with M (Figure S5). In addition, DLSm was preferred over DLSu in most OARs, except for the spinal cord, pharynx, and larynx; furthermore, DLSm was preferred over DIR for all OARs. 

Quality assurance for review purposes suggests relatively low rates of major errors for M, DLSm, and DLSu, accounting for 1.9%, 2.7%, and 4.2%, respectively, in contrast to 38.1% for DIR (Figure 5C). In addition, DLSm needs both minor and major revisions of contours less often than DLSu does (28.0% vs. 38.4%), and this difference was significant in the submandibular gland, parotid gland, pharynx, esophagus, and spinal cord (Figure S5). Moreover, the rate of revision required was comparable between M and DLSm (overall, 29.7% vs. 28.0%) except for the larynx (44.2% vs. 56.2%). 

## 4. Discussion

Although several studies regarding DLS in RT planning have been reported recently [12], the feasibility of DLS in ART and an ideal training method for DLS have not been reported yet. In the current study, based on both quantitative and subjective measurements, we demonstrated the feasibility of DLS and the importance of continual development in DLS with individualized training sets in ART for H&N cancer. 

Patients with H&N cancer frequently experience dry mouth, sore throat, and taste changes during RT, which negatively affects the oral intake of patients, resulting in significant weight loss [25]. In addition to weight loss, volumetric shrinkage [26,27] or migration of normal organs [28] could hamper the accurate delivery of the initially planned RT dose to patients [29,30]. This implies that additional work for re-planning would be required over the course of treatment. That is, the so-called ART is needed to compensate for these structural changes [29,30]. Currently, a fixed-term ART, which is highly dependent on physicians’ discretion or departments’ resources, is frequently performed in several centers owing to the time-consuming RT planning process [30]. The more accurate segmentation results of DLSm, compared to those of DLSu and DIR, in the current study following a volumetric reduction in the glandular structures, may enable either real-time or short-term ART. Several previous papers [31,32,33] have reported volume reduction in the parotid or submandibular glands by 12–35% or 1.1–1.5% per day during RT, which is consistent with the current results. Consequently, the improved FPD in DLSm can potentially prevent unnecessary target coverage compromises arising from the over-segmentation of contracted OARs. In addition, a majority (72.0%) of the results obtained from continual training (DLSm) were deemed satisfactory by the experts, which indicates the robustness of DLSm compared with DLSu (61.6%) or DIR (25.9%), as well as inter-observer acceptability comparable with that of M (71.6%). 

The improvement of the results from continual training compared with the results of previous studies on DLS using conventional training for the H&N region was nuanced but measurable (Table 3). Although the number of training sets (80 sets) was relatively low compared with that in the recent work by van Dijk et al. [13], both DLSm and DLSu exhibited a higher DSC concerning the esophagus, pharynx, larynx, and glandular structures. The two-step approach employed in the DLS algorithm in the current study would theoretically improve the overall accuracy despite the limited number of training sets. A similar approach was introduced by Liang et al. [34]: the use of a bounding box around OARs, followed by segmentation within the box. They reported an overall DSC of 0.86, better than that obtained in the present study (0.81); an increased number of training samples (185 sets) could explain the robustness of their DLS. The impact of the training sample size has been reported by Fang et al. [35], who demonstrated that DLS based on data obtained from 800 patients achieved more accurate results compared with that based on data from 200 patients. However, the impact of continual training with regard to DLS on ART segmentation has not been investigated yet. It has been reported that the use of different training datasets with the same network could lead to different results [36]. Furthermore, the continuous training and refinement of DLS could guarantee improved performance regarding both objective and subjective measures. The impact of continual training in DLS on dosimetric outcomes needs to be investigated in future work. 

Unlike widely adopted atlas-based auto-segmentation, which propagates libraries from multiple patients to the subject image [12], simple DIR merges the single contour from an individual patient to the test set. In the current study, DIR achieved a suboptimal average DSC of 0.70 with substantially increased FPD, HD, and MSD. Moreover, most observers determined 38.1% of DIR to be edited with a major error. Mencarelli et al. [48] reported the limitation of adopting DIR for contouring in the H&N region with random errors of 2.2 and 3.3 mm for OARs and tumors, respectively. Although the accuracy evaluated based on the distance-to-agreement criterion could underestimate the performance of DIR owing to the variability of human-derived ground truth and registration landmarks [49], the subjective judgment of experts in active clinical practice also discouraged the application of DIR compared with that of DLS in the current study. 

Although we performed multiple quantitative assessments, including assessments of similarity (DSC), classification (FPD, FND), and distance-to-agreement (MSD, HD), these metrics could overestimate the overall accuracy of segmentation. This is because the baseline “ground-truth” may not be the exact answer owing to its inter-observer variability and uncertainty [4,5,6]. In the current study, observers responded that 29.7% of M might need to be modified, demonstrating the inter-observer variability. Subjective assessment using the Turing test would allow the evaluation of the acceptance level for each individual, mitigate institutional bias, and help determine the degree of human-level performance [13,24]. Although the difference between DLSm and DLSu was significant but subtle in terms of quantitative metrics, subjective assessments revealed that most clinicians valued DLSm over DLSu in the ART setting. Therefore, the grading of segmentation performance should be performed via both objective and subjective assessments in future investigations regarding segmentation.

High-quality training set rather than low-quality but large-volume training set emerges as a simple but effective approach for improving the performance of DLS. Zhao et al. proposed synthetic CT generation for training DLS from extremely limited training set [50]. They generated up to 2000 synthetic CT from 30 well-defined segmentations for training DLS resulting in DSC of 0.74–0.83 [50]. Currently, various DIR software is recommended for ART; DLS is considered as a potential next step in near future [1,51]. In this context, the current method of continual training with initial planning CT (DLSm) for ART could represent a high-quality training data acquisition. Further investigations need to be conducted to determine whether DLSm could be applicable in the real clinical practice. 

Some limitations of the current study should be acknowledged. First, although 100 patients were randomly selected and had well-balanced baseline characteristics, there remains a selection bias in terms of CT samples. Second, the lack of external validation based on CT data from other institutions hinders further implementations in clinical practice. Nevertheless, we hypothesized the potential benefit of continual training on an individualized (matched) training set for DLS in ART for the H&N region. The results of the present study supported this hypothesis, and they were preliminarily validated using the Turing test based on the expert opinions of multi-institutional physicians. Regarding manual segmentation, 29.7% of respondents disagreed to contouring by a single physician. However, most disagreement results from <10% of contour volume (27.8% for minor error with revision) which was consistent with underlying contouring variability among observers, as previously reported [7,8,9]. Since the current training set was based on manual segmentation by a single physician, the inter-physician variability for OARs in constructing training set needs to be considered in the next phase. In addition, the dismal results obtained for small organs (i.e., cochlea, temporomandibular joint, optic nerve, and optic chiasm) could be owing to the limitations of CT such as relatively poor tissue resolution, which could be improved by performing the segmentation based on MRI. Based on this preliminary study of DLS in ART for the H&N region, further investigations could evaluate the dosimetric and clinical impact of DLSm based on continual training with an individualized training set based on daily kilovoltage or megavoltage cone-beam CT during fractionated RT. We only included OARs rather than gross tumor volume or clinical target volume following reasons. Firstly, CT-based delineation is hard to define the accurate extent of tumor. Secondly, an inter-observer variation of tumor volume usually surpasses that of OARs. However, a future study incorporating tumor volumes is needed to assess the continual training in the real clinical practice.

## 5. Conclusions

In conclusion, we observed the effectiveness of DLS for OARs in the H&N region. According to our results, DLS outperformed DIR in terms of both objective and subjective metrics. In addition, DLS achieved human-level performance within the range of interobserver variability. In addition, the refinement and continual training of already built DLS models could provide better optimization and guarantee robustness compared with fixed DLS based on data obtained from independent patients when personalized ART is needed. 

After future studies with consistent results supporting continual training, it is suggested for researchers to develop DLS software with continual training for ART to optimize the outcomes.

## Figures and Tables

**Table 1:** Average volumetric Dice coefficient, false positive Dice coefficient, and false negative Dice coefficient for, deep learning-based segmentation from unmatched set (DLSu), matched training set (DLSm), and contouring from deformable image registration of primary planning computed tomography (DIR).

	Volumetric Dice Coefficient			False Positive Dice Coefficient			False Negative Dice Coefficient		
	DLSu	DLSm	DIR	DLSu	DLSm	DIR	DLSu	DLSm	DIR
	Mean ± SD	Mean ± SD	Mean ± SD	Mean ± SD	Mean ± SD	Mean ± SD	Mean ± SD	Mean ± SD	Mean ± SD
**All**	0.80 ** ± 0.02	0.83 * ± 0.02	0.70 ^†^ ± 0.05	0.19 ** ± 0.03	0.19 ± 0.03	0.33 ^†^ ± 0.07	0.20 ** ± 0.04	0.18 * ± 0.03	0.28 ^†^ ± 0.05
**Central organs**									
Brainstem	0.87 ± 0.02	0.87 ± 0.03	0.87 ± 0.05	0.18 ± 0.07	0.19 ± 0.09	0.15 ^†^ ± 0.10	0.07 ** ± 0.03	0.07 ± 0.04	0.12 ^†^ ± 0.06
Spinal cord	0.82 ** ± 0.04	0.82 ± 0.04	0.67^†^ ± 0.16	0.15 ** ± 0.10	0.17 ± 0.10	0.33 ^†^ ± 0.21	0.21 ± 0.12	0.18 ± 0.11	0.33 ^†^ ± 0.19
Esophagus	0.80 ± 0.07	0.82 ± 0.04	0.74 ± 0.10	0.20 ± 0.09	0.22 ± 0.07	0.28 ± 0.14	0.20 ± 0.14	0.13 * ± 0.06	0.25 ^†^ ± 0.10
Oral cavity	0.91 ± 0.02	0.91 ± 0.02	0.88 ^†^ ± 0.04	0.11 ± 0.06	0.09 * ± 0.05	0.12 ± 0.08	0.07 ± 0.04	0.08 ± 0.04	0.11 ± 0.07
Pharynx	0.82 ** ± 0.03	0.82 ± 0.03	0.73 ^†^ ± 0.11	0.20 ** ± 0.08	0.28 * ± 0.09	0.29 ± 0.13	0.15 ** ± 0.07	0.08 * ± 0.05	0.26 ^†^ ± 0.14
Larynx	0.85 ** ± 0.05	0.85 ± 0.04	0.77 ^†^ ± 0.09	0.20 ± 0.12	0.19 ± 0.13	0.26 ± 0.17	0.09 ** ± 0.10	0.11 ± 0.10	0.20 ^†^ ± 0.12
**Bony structures**									
Mandible	0.95 ** ± 0.01	0.95 ± 0.01	0.85 ^†^ ± 0.09	0.03 ** ± 0.02	0.05 * ± 0.02	0.15 ^†^ ± 0.10	0.07 ** ± 0.03	0.05 * ± 0.03	0.15 ^†^ ± 0.09
R_cochlea	0.76 ± 0.07	0.76 ± 0.08	0.68 ± 0.15	0.32 ± 0.11	0.21 * ± 0.09	0.34 ^†^ ± 0.19	0.17 ± 0.12	0.26 ± 0.15	0.29 ± 0.20
L_cochlea	0.73 ± 0.07	0.76 ± 0.07	0.71 ± 0.14	0.32 ± 0.16	0.25 ± 0.13	0.31 ± 0.15	0.22 ± 0.13	0.24 ± 0.16	0.28 ± 0.22
R_TMJ	0.72 ± 0.07	0.70 ± 0.08	0.65 ± 0.14	0.25 ± 0.10	0.25 ± 0.13	0.31 ± 0.20	0.30 ± 0.17	0.35 ± 0.18	0.39 ± 0.19
L_TMJ	0.74 ± 0.07	0.75 ± 0.05	0.71 ± 0.11	0.27 ± 0.13	0.21 ± 0.11	0.24 ± 0.15	0.26 ± 0.10	0.29 ± 0.14	0.34 ± 0.16
**Glandular structures**									
R_parotidG	0.85 ** ± 0.04	0.87 * ± 0.03	0.76 ^†^ ± 0.08	0.17 ** ± 0.08	0.13 ± 0.06	0.34 ^†^ ± 0.13	0.14 ± 0.08	0.13 ± 0.06	0.14 ± 0.08
L_parotidG	0.84 ** ± 0.04	0.86 * ± 0.02	0.77 ^†^ ± 0.07	0.18 ** ± 0.07	0.12 * ± 0.05	0.32 ^†^ ± 0.13	0.13 ± 0.06	0.15 ± 0.06	0.15 ± 0.08
R_SMG	0.81 ** ± 0.10	0.88 * ± 0.04	0.71 ^†^ ± 0.09	0.06 ** ± 0.03	0.10 * ± 0.04	0.40 ^†^ ± 0.13	0.32 ** ± 0.21	0.15 * ± 0.08	0.19 ± 0.11
L_SMG	0.83 ** ± 0.06	0.86 * ± 0.04	0.71^†^ ± 0.11	0.07 ** ± 0.04	0.10 * ± 0.05	0.39 ^†^ ± 0.14	0.28 ± 0.12	0.17 * ± 0.08	0.19 ± 0.14
Thyroid	0.88 ** ± 0.08	0.88 ± 0.04	0.70^†^ ± 0.14	0.10 ** ± 0.04	0.10 ± 0.05	0.33 ^†^ ± 0.15	0.15 ** ± 0.16	0.14 ± 0.08	0.27 ^†^ ± 0.17
**Optic apparatus**									
R_eye	0.91 ** ± 0.02	0.92 ± 0.02	0.84 ^†^ ± 0.06	0.12 ± 0.06	0.09 * ± 0.06	0.16 ^†^ ± 0.07	0.05 ** ± 0.03	0.07 * ± 0.04	0.16 ^†^ ± 0.09
L_eye	0.91 ** ± 0.02	0.90 ± 0.02	0.83 ^†^ ± 0.07	0.09 ** ± 0.07	0.13 * ± 0.08	0.18 ± 0.09	0.09 ** ± 0.06	0.06 * ± 0.05	0.16 ^†^ ± 0.11
R_lens	0.78 ** ± 0.08	0.79 ± 0.09	0.52 ^†^ ± 0.22	0.32 ** ± 0.17	0.27 ± 0.16	0.54 ^†^ ± 0.32	0.11 ** ± 0.10	0.15 ± 0.10	0.42 ^†^ ± 0.22
L_lens	0.76 ** ± 0.13	0.78 ± 0.09	0.45 ^†^ ± 0.24	0.22 ** ± 0.20	0.28 ± 0.19	0.63 ^†^ ± 0.33	0.26 ** ± 0.27	0.16 ± 0.14	0.47 ^†^ ± 0.25
R_optic nerve	0.72 ** ± 0.07	0.70 ± 0.07	0.58 ^†^ ± 0.14	0.22 ** ± 0.10	0.16 * ± 0.09	0.36 ^†^ ± 0.18	0.34 ** ± 0.13	0.44 * ± 0.11	0.49 ± 0.17
L_optic nerve	0.70 ** ± 0.07	0.72 ± 0.07	0.57 ^†^ ± 0.15	0.17 ** ± 0.07	0.16 ± 0.07	0.36 ^†^ ± 0.17	0.43 ± 0.13	0.40 ± 0.15	0.49 ± 0.19
Optic chiasm	0.53 ** ± 0.16	0.52 ± 0.17	0.35 ^†^ ± 0.21	0.48 ** ± 0.21	0.64 * ± 0.20	0.78 ± 0.25	0.46 ± 0.24	0.31 * ± 0.21	0.51 ^†^ ± 0.28

Footnotes: Statistically significant difference (*p* value of < 0.0167) after the Wilcoxon signed rank test between DLSm and DLSu (*), between DLSm and DIR (^†^), and between DLSu and DIR (**). Abbreviations: SD, standard deviation; R, right; L, left; TMJ, temporomandibular joint; parotidG, parotid gland; SMG, submandibular gland.

**Table 2:** Average Hausdorff distance and mean surface distance for deep learning-based segmentation for unmatched set (DLSu), matched training set (DLSm), and contouring from deformable image registration of primary planning computed tomography (DIR).

	Hausdorff Distance (mm)			Mean Surface Distance (mm)		
	DLSu	DLSm	DIR	DLSu	DLSm	DIR
	Mean ± SD	Mean ± SD	Mean ± SD	Mean ± SD	Mean ± SD	Mean ± SD
**All**	3.04 ** ± 0.36	2.79 * ± 0.22	4.19 ^†^ ± 0.74	1.05 ** ± 0.10	0.98 * ± 0.07	1.61 ^†^ ± 0.31
**Central organs**						
Brainstem	2.96 ± 0.34	3.13 ± 0.43	3.25 ± 0.86	1.20 ** ± 0.22	1.29 ± 0.28	1.26 ^†^ ± 0.43
Spinal cord	2.09 ** ± 0.48	2.10 ± 0.50	3.97 ^†^ ± 2.15	0.84 ** ± 0.24	0.82 ± 0.21	1.56 ^†^ ± 0.77
Esophagus	3.66 ± 2.15	3.04 ± 0.88	4.20 ± 1.44	1.28 ** ± 0.53	1.12 ± 0.23	1.62 ^†^ ± 0.58
Oral cavity	4.60 ± 1.42	4.28 ± 0.94	5.75 ± 2.71	1.70 ± 0.42	1.59 ± 0.30	2.15 ^†^ ± 0.93
Pharynx	3.53 ** ± 0.84	3.53 ± 0.52	5.18 ^†^ ± 2.05	1.39 ** ± 0.26	1.44 ± 0.20	2.01 ^†^ ± 0.80
Larynx	4.19 ** ± 1.48	4.26 ± 1.34	6.54 ^†^ ± 2.39	1.61 ** ± 0.54	1.66 ± 0.52	2.54 ^†^ ± 1.06
**Bony structures**						
Mandible	1.28 ** ± 0.27	1.27 ± 0.37	3.55 ^†^ ± 2.64	0.48 ± 0.12	0.47 ± 0.09	1.31 ± 0.87
R_cochlea	2.36 ± 0.60	2.26 ± 0.52	2.70 ± 0.89	0.74 ± 0.22	0.70 ± 0.22	0.97 ± 0.43
L_cochlea	2.61 ± 0.53	2.40 ± 0.67	2.47 ± 0.66	0.83 ± 0.19	0.73 ± 0.19	0.88 ± 0.39
R_TMJ	3.56 ± 1.27	4.13 ± 1.53	4.39 ± 1.99	1.22 ± 0.44	1.36 ± 0.44	1.55 ± 0.71
L_TMJ	3.29 ± 0.86	3.36 ± 1.14	3.61 ± 1.34	1.17 ± 0.31	1.14 ± 0.30	1.29 ± 0.54
**Glandular structures**						
R_parotidG	3.91 ± 1.09	3.16 * ± 0.41	5.36 ± 2.27	1.41 ** ± 0.33	1.18 * ± 0.18	2.25 ^†^ ± 0.97
L_parotidG	3.78 ± 0.66	3.32 * ± 0.61	5.08 ± 2.02	1.43 ** ± 0.22	1.25 * ± 0.16	2.17 ^†^ ± 0.83
R_SMG	4.01 ± 2.18	2.45 * ± 0.78	5.03 ± 1.80	1.30 ** ± 0.64	0.84 * ± 0.22	2.09 ^†^ ± 0.73
L_SMG	3.60 ** ± 1.15	2.72 * ± 0.82	4.99 ^†^ ± 1.75	1.20 ** ± 0.38	0.96 * ± 0.29	2.08 ^†^ ± 0.83
Thyroid	2.56 ** ± 2.57	2.28 ± 0.89	4.83 ^†^ ± 1.90	0.84 ** ± 0.58	0.76 ± 0.17	1.88 ^†^ ± 0.79
**Optic apparatus**						
R_eye	2.05 ** ± 0.40	1.94 ± 0.38	3.11 ^†^ ± 0.73	0.72 ** ± 0.14	0.68 ± 0.14	1.25 ^†^ ± 0.45
L_eye	2.12 ** ± 0.42	2.13 ± 0.53	3.53 ^†^ ± 1.17	0.75 ± 0.13	0.78 ± 0.19	1.36 ^†^ ± 0.58
R_lens	1.90 ** ± 0.90	1.71 ± 0.84	3.41 ^†^ ± 1.47	0.59 ** ± 0.22	0.56 ± 0.23	1.40 ^†^ ± 0.75
L_lens	1.85 ** ± 0.93	1.94 ± 0.99	4.15 ^†^ ± 2.01	0.63 ** ± 0.32	0.59 ± 0.22	1.75 ^†^ ± 1.05
R_optic nerve	2.74 ± 1.30	2.57 ± 0.86	3.43 ± 1.02	0.74 ** ± 0.25	0.74 ± 0.17	1.07 ^†^ ± 0.37
L_optic nerve	3.58 ± 3.09	2.44 ± 0.74	3.57 ± 1.09	0.91 ** ± 0.50	0.71 ± 0.20	1.11 ^†^ ± 0.39
Optic chiasm	3.64 ± 0.95	3.67 ± 0.93	4.25 ± 1.46	1.18 ** ± 0.38	1.24 ± 0.40	1.57 ^†^ ± 0.50

Footnotes: Statistically significant difference (*p* value of < 0.0167) after the Wilcoxon signed rank test between DLSm and DLSu (*), between DLSm and DIR (^†^), and between DLSu and DIR (**). Abbreviations: SD, standard deviation; R, right; L, left; TMJ, temporomandibular joint; parotidG, parotid gland; SMG, submandibular gland.

**Table 3:** Average volumetric Dice coefficient of our model and for previously published results.

	Brain Stem	Spinal Cord	Esophagus	Pharynx	Larynx	Mandible	Cochlea
Current, DLSu	0.87	0.82	0.80	0.82	0.85	0.95	0.75
Current, DLSm	0.87	0.82	0.82	0.82	0.85	0.95	0.76
Fritscher et al. [37]							
Ibragimov et al. [38]		0.87					
Mocnik et al. [39]							
Ren X et al. [40]							
Zhu et al. [41]	0.87					0.93	
Nikolov et al. [36]	0.84	0.88				0.94	0.70
Tong et al. [42]	0.87					0.94	
van Rooij et al. [43]	0.64		0.60	0.71	0.78		
Rhee et al. [44]	0.86	0.83	0.81			0.87	0.66
Liang et al. [34]	0.90	0.88			0.87	0.91	0.82
van Dijk et al. [13]	0.84	0.87	0.55	0.68	0.71	0.94	
Wong et al. [45]	0.80–0.83	0.79					
Zhensong et al. [46]	0.90					0.94	
Oktay et al. [47]	0.79–0.90	0.82–0.93				0.94–0.99	
	**ParotidG**	**SMG**	**Thyroid**	**Eye**	**Lens**	**Optic nerve**	**Optic chiasm**
Current, DLSu	0.85	0.82	0.88	0.91	0.77	0.71	0.53
Current, DLSm	0.87	0.87	0.88	0.91	0.79	0.71	0.52
Fritscher et al. [37]	0.81	0.65					0.51
Ibragimov et al. [38]	0.78	0.71		0.88		0.64	0.37
Mocnik et al. [39]	0.79						
Ren X et al. [40]						0.71	0.58
Zhu et al. [41]	0.87	0.81				0.71	0.53
Nikolov et al. [36]	0.86	0.77		0.95	0.80	0.70	
Tong et al. [42]	0.83	0.78				0.67	0.58
van Rooij et al. [43]	0.83	0.82					
Rhee et al. [44]	0.83			0.89	0.72	0.69	0.41
Liang et al. [34]	0.85				0.84	0.69	
van Dijk et al. [13]	0.84	0.78	0.83				
Wong et al. [45]	0.80	0.81–0.82		0.85–0.88		0.43–0.47	0.32–0.38
Zhensong et al. [46]	0.83						
Oktay et al. [47]	0.83–0.93	0.75–0.92		0.92–0.97			

Abbreviations: parotidG, parotid gland; SMG, submandibular gland; DLSu, deep learning-based segmentation using unmatched training set; DLSm, deep learning-based segmentation using matched training set.

## Data Availability

Data availability is limited due to institutional data protection law and confidentiality of patient data.

## Supplementary Materials

The following are available online at https://www.mdpi.com/2072-6694/13/4/702/s1, Figure S1: Median and interquartile range of average Dice coefficient (DSC), false positive Dice (FPD), false negative Dice (FND), hausdorff distance (HD), and mean surface distance (MSD) for central organs. Figure S2: Median and interquartile range of average dice coefficient (DSC), false positive dice (FPD), false negative dice (FND), hausdorff distance (HD), and mean surface distance (MSD) for bony structures. Figure S3: Median and interquartile range of average dice coefficient (DSC), false positive dice (FPD), false negative dice (FND), hausdorff distance (HD), and mean surface distance (MSD) for glandular structures. Figure S4: Median and interquartile range of average dice coefficient (DSC), false positive dice (FPD), false negative dice (FND), hausdorff distance (HD), and mean surface distance (MSD) for optic apparatus. Figure S5: Detailed results of subjective evaluation using Turing test. The rate of discrimination of a single contour as a human (A); comparison between two contours (B); quality assurance for review purpose of a single contour (C). Table S1: Lists of organ-at-risk according to four subgroups. Table S2: Patient and tumor characteristics of training and test set. Table S3: Volumetric changes of contour between primary planning computed tomography (CT) and adaptive planning CT.
