# Supplementary material for: Feasibility of Continual Deep Learning-Based Segmentation for Personalized Adaptive Radiation Therapy in Head and Neck Area
Source: Cancers (Basel). 2021 Feb 9;13(4):702. doi: 10.3390/cancers13040702 (PMC7915955; doi:10.3390/cancers13040702)
Supplement: Supplementary file 1 [file cancers-13-00702-s001.pdf]

cancers

Supplementary materials: Feasibility of Continual Deep Learning-Based Segmentation for Personalized Adaptive Radiation Therapy in Head and Neck Area

Nalee Kim, Jaehee Chun, Jee Suk Chang, Chang Geol Lee, Ki Chang Keum and Jin Sung Kim

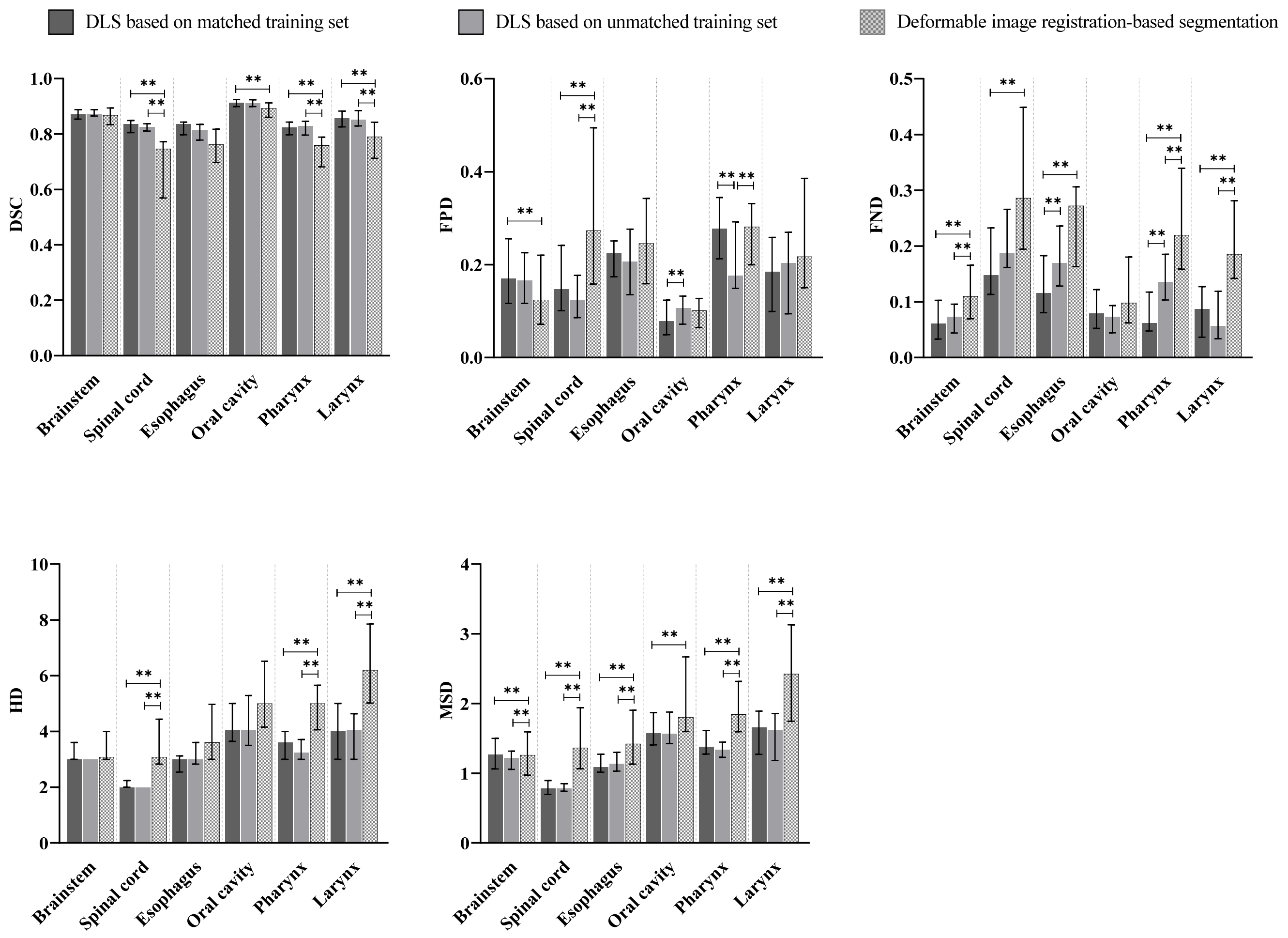

Figure S1. Median and interquartile range of average Dice coefficient (DSC), false positive Dice (FPD), false negative Dice (FND), hausdorff distance (HD), and mean surface distance (MSD) for central organs. (** indicates statistical significance).

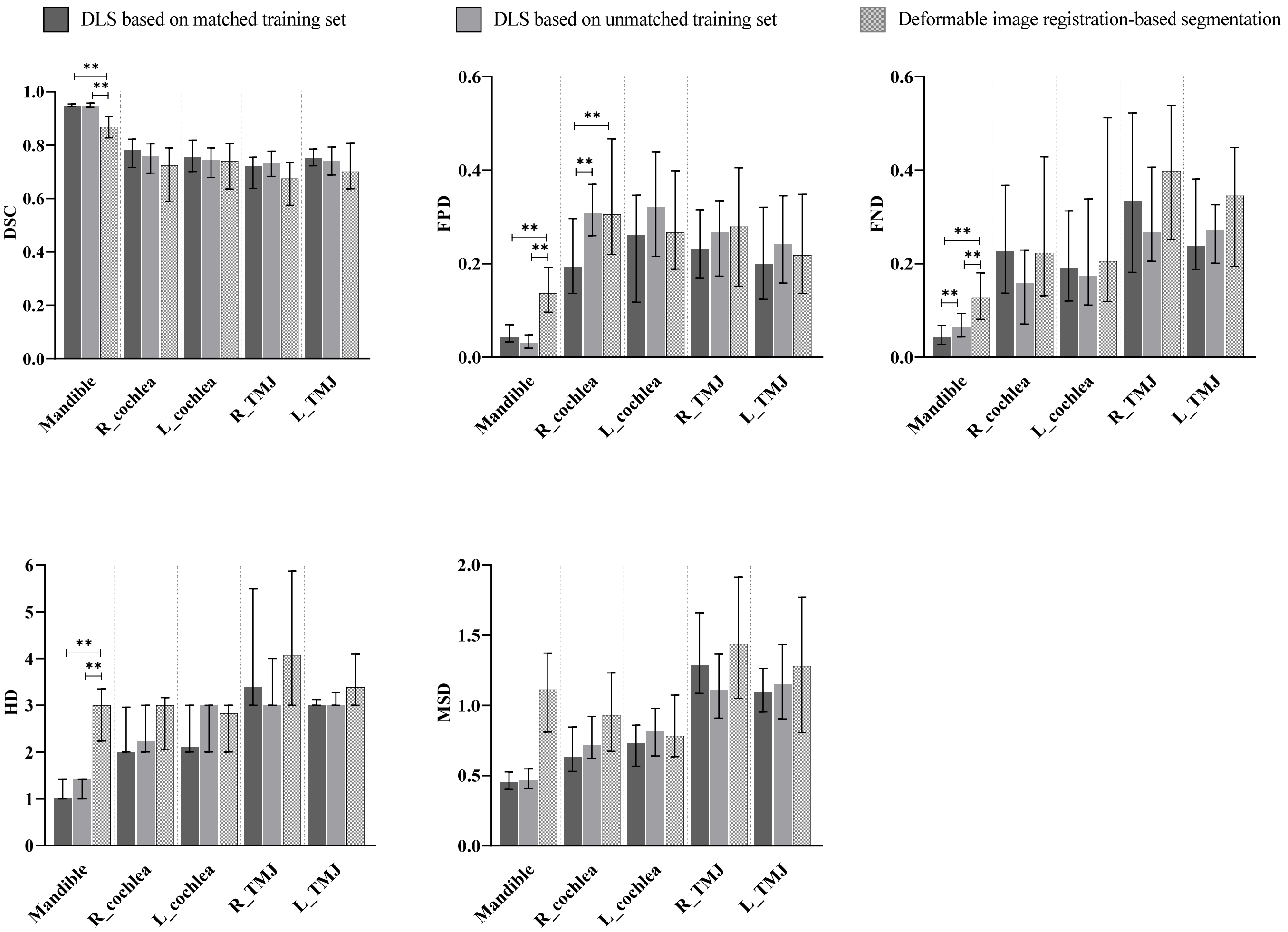

Figure S2. Median and interquartile range of average dice coefficient (DSC), false positive dice (FPD), false negative dice (FND), hausdorff distance (HD), and mean surface distance (MSD) for bony structures. (** indicates statistical significance). **Abbreviations:** R, right; L, left; TMJ, temporomandibular joint.

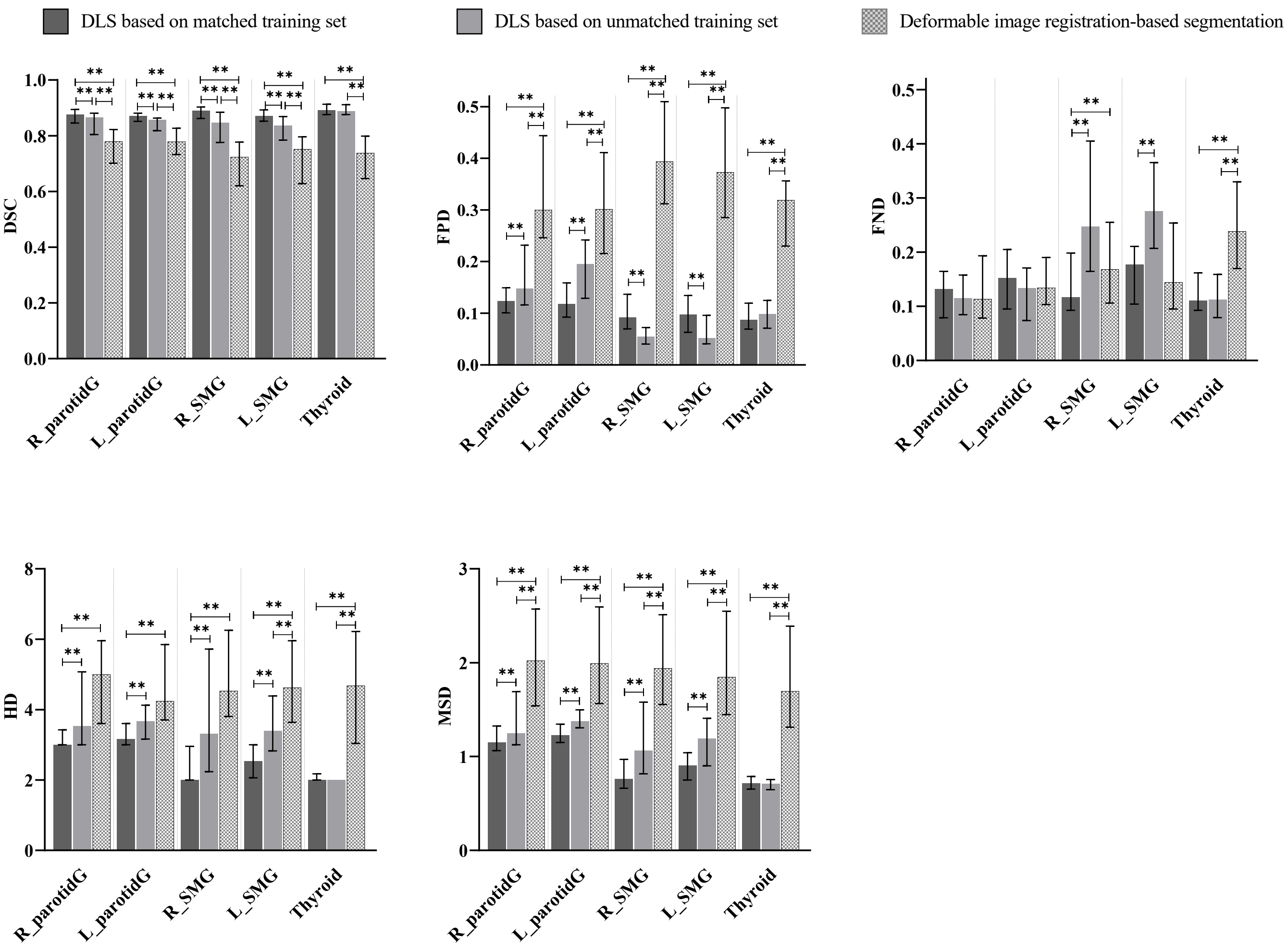

Figure S3. Median and interquartile range of average dice coefficient (DSC), false positive dice (FPD), false negative dice (FND), hausdorff distance (HD), and mean surface distance (MSD) for glandular structures. (** indicates statistical significance). **Abbreviations:** R, right; L, left; parotidG, parotid gland; SMG, submandibular gland.

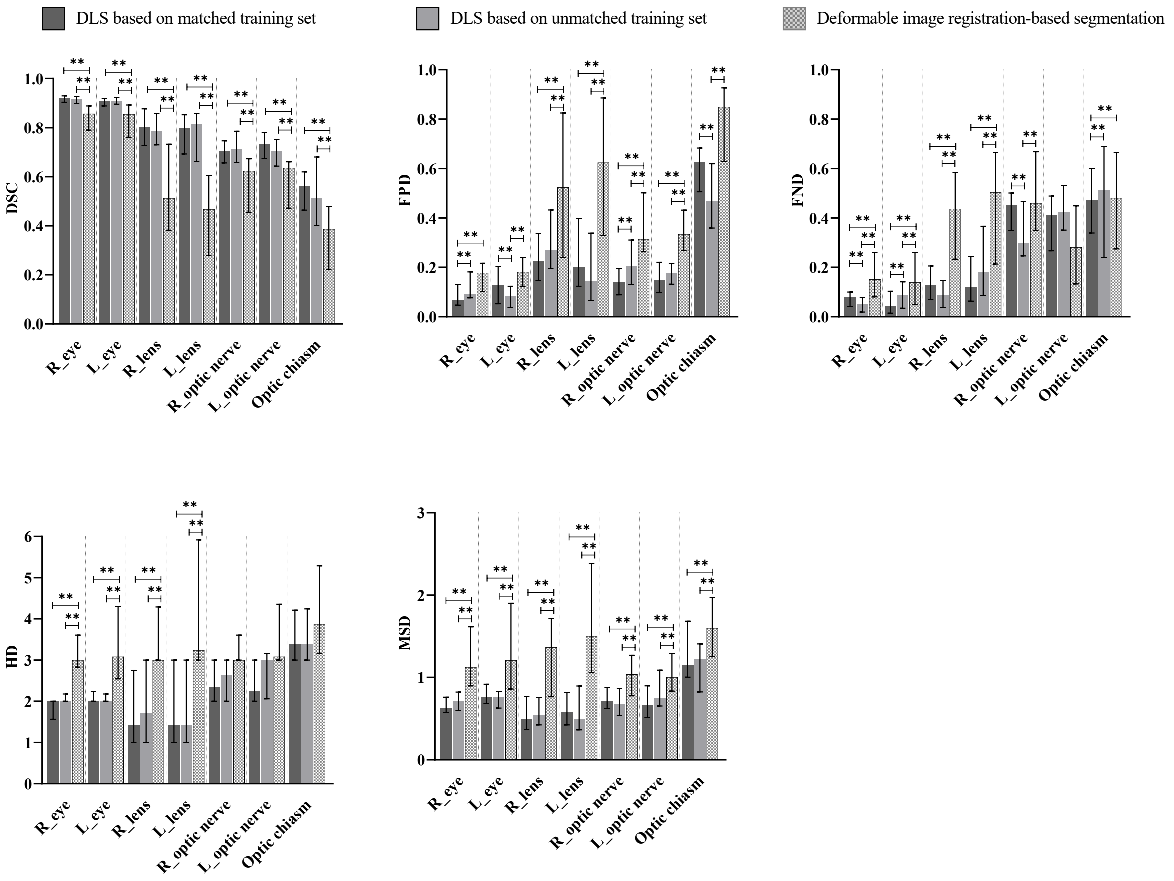

Figure S4. Median and interquartile range of average dice coefficient (DSC), false positive dice (FPD), false negative dice (FND), hausdorff distance (HD), and mean surface distance (MSD) for optic apparatus. **Abbreviations:** R, right; L, left.

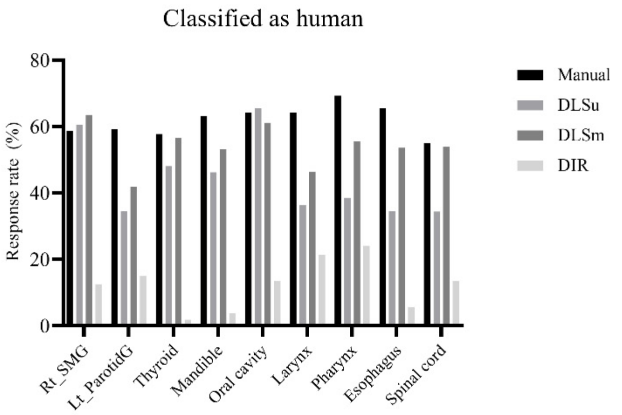

	Manual	DLSu	DLSm	DIR
Rt_SMG	58.7	60.5	63.5	12.5
Lt_ParotidG	59.2	34.6	41.8	15.0
Thyroid	57.7	48.1	56.6	1.8
Mandible	63.1	46.2	53.1	3.8
Oral cavity	64.2	65.4	61.1	13.5
Larynx	64.3	36.3	46.4	21.3
Pharynx	69.3	38.5	55.6	24.1
Esophagus	65.4	34.6	53.6	5.6
Spinal cord	55.0	34.4	53.9	13.5

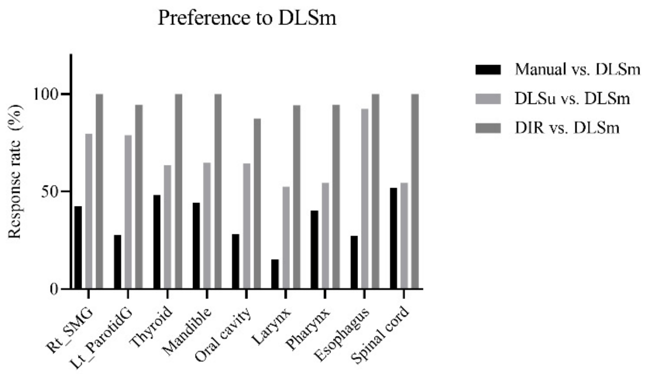

	Manual vs. DLSm	DLSu vs. DLSm	DIR vs. DLSm
Rt_SMG	42.5	79.7	100.0
Lt_ParotidG	27.6	78.9	94.5
Thyroid	48.1	63.5	100.0
Mandible	44.2	64.7	100.0
Oral cavity	28.2	64.5	87.4
Larynx	15.3	52.5	94.3
Pharynx	40.4	54.4	94.5
Esophagus	27.2	92.4	100.0
Spinal cord	51.9	54.4	100.0

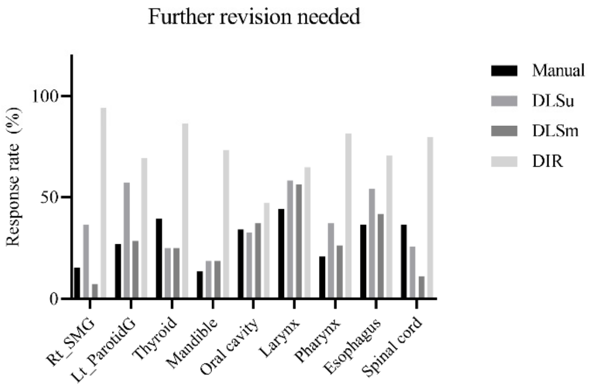

	Manual	DLSu	DLSm	DIR
Rt_SMG	15.4	36.5	7.3	94.3
Lt_ParotidG	27.0	57.0	28.5	69.2
Thyroid	39.4	25.0	25.0	86.6
Mandible	13.5	18.7	18.6	73.3
Oral cavity	34.1	32.7	37.2	47.1
Larynx	44.2	58.3	56.2	64.7
Pharynx	20.8	37.3	26.4	81.3
Esophagus	36.5	54.1	41.9	70.4
Spinal cord	36.5	25.7	11.2	79.6

Figure S5. Detailed results of subjective evaluation using Turing test. The rate of discrimination of a single contour as a human (A); comparison between two contours (B); quality assurance for review purpose of a single contour (C).

Table S1. Lists of organ-at-risk according to four subgroups.

List of organ-at-risk			
**Central organs**	**Bony structures**	**Glandular structures**	**Optic apparatus**
Brainstem	Mandible	R_parotidG	R_eye
Spinal cord	R_cochlea	L_parotidG	L_eye
Esophagus	L_cochlea	R_SMG	R_lens
Oral cavity	R_TMJ	L_SMG	L_lens
Pharynx	L_TMJ	Thyroid	R_optic nerve
Larynx			L_optic nerve
			Optic chiasm

Abbreviations: R, right; L, left; TMJ, temporomandibular joint; parotidG, parotid gland; SMG, submandibular gland.

Table S2. Patient and tumor characteristics of training and test set.

	**Training set for unmatched patients**	**Training set for matched patients**		**Test set**	
	**N = 80**	**N = 80**	**P-value***	**N = 20**	**P-value****
	**N (%)**	**N (%)**		**N (%)**	
Sex			1.000		1.000
Male	69 (86.3)	69 (86.3)		18 (90.0)	
Female	11 (13.8)	11 (13.8)		2 (10.0)	
Primary			0.807		
Nasopharynx	36 (45.0)	33 (41.3)		8 (40.0)	0.916
Oropharynx	27 (33.8)	26 (32.5)		6 (30.0)	
Hypopharynx	17 (21.2)	21 (26.2)		6 (30.0)	
Staging			0.661		0.929
T1	20 (25.0)	26 (32.5)		6 (30.0)	
T2	20 (25.0)	21 (26.3)		6 (30.0)	
T3	26 (32.5)	20 (25.0)		5 (25.0)	
T4	14 (17.5)	13 (16.3)		3 (15.0)	
Node metastasis			0.968		0.989
N0	12 (15.0)	14 (17.5)		2 (10.0)	
N1	30 (37.5)	31 (38.8)		9 (45.0)	
N2	32 (40.0)	29 (36.3)		8 (40.0)	
N3	6 (7.5)	6 (7.5)		1 (5.0)	

Note: Comparison between training set for matched patients and unmatched patients (*) and among both training sets and test set (**).

Table S3. Volumetric changes of contour between primary planning computed tomography (CT) and adaptive planning CT.

	Primary CT		Adaptive CT		P-value
	**Median**	**[IQR]**	**Median**	**[IQR]**	
Central organs					
Brainstem	32.9	[31.0;35.3]	31.5	[29.6;33.8]	0.455
Spinal cord	20.2	[17.8;22.2]	19.1	[17.6;23.0]	0.985
Esophagus	22.9	[19.3;25.5]	19.9	[17.8;24.9]	0.261
Oral cavity	147.0	[133.1;200.3]	151.8	[137.8;186.5]	0.812
Pharynx	62.3	[54.5;73.9]	61.2	[53.4;69.3]	0.216
Larynx	43.0	[38.7;49.2]	40.2	[37.1;46.8]	0.165
Bony structures					
Mandible	115.5	[97.4;133.5]	114.2	[95.8;132.5]	0.452
R_cochlea	0.8	[0.7;1.0]	0.8	[0.7;0.8]	0.185
L_cochlea	0.8	[0.6;1.0]	0.7	[0.6;0.9]	0.881
R_TMJ	2.8	[2.6;3.5]	3.2	[2.8;3.6]	0.261
L_TMJ	3.3	[2.4;3.9]	3.3	[3.0;3.8]	0.330
Glandular structures					
R_parotidG	32.5	[25.1;43.7]	27.3	[21.1;34.1]	<0.001
L_parotidG	31.8	[24.8;43.4]	28.9	[21.0;33.8]	0.001
R_SMG	12.5	[11.6;15.7]	10.7	[8.9;12.2]	<0.001
L_SMG	13.0	[11.4;14.7]	11.5	[9.4;12.1]	<0.001
Thyroid	25.3	[17.5;30.3]	23.0	[17.3;29.0]	0.202
Optic apparatus					
R_eye	10.3	[9.3;11.5]	10.6	[9.9;11.1]	0.956
L_eye	10.5	[9.7;11.8]	10.5	[9.7;11.4]	0.522
R_lens	0.4	[0.3;0.5]	0.4	[0.3;0.4]	0.104
L_lens	0.4	[0.4;0.5]	0.4	[0.3;0.4]	0.104
R_optic nerve	0.9	[0.8;1.1]	1.1	[1.0;1.2]	0.040
L_optic nerve	1.0	[0.8;1.1]	1.1	[1.0;1.2]	0.040
Optic chiasm	0.7	[0.7;0.9]	0.7	[0.5;0.8]	0.020

Abbreviations: IQR, interquartile range; R, right; L, left; TMJ, temporomandibular joint; parotidG, parotid gland; SMG, submandibular gland.
